# Effects of NCO/OH Ratios on Bio-Based Polyurethane Film Properties Made from *Acacia mangium* Liquefied Wood

**DOI:** 10.3390/polym15051154

**Published:** 2023-02-24

**Authors:** Ismawati Palle, Valeritta Lodin, Ag Ahmad Mohd Yunus, Seng Hua Lee, Paridah Md Tahir, Naruhito Hori, Petar Antov, Akio Takemura

**Affiliations:** 1 Faculty of Tropical Forestry, Universiti Malaysia Sabah, Jalan UMS, Kota Kinabalu 88400, Sabah, Malaysia; 2 Department of Wood Industry, Faculty of Applied Sciences, University Teknologi MARA (UiTM) Cawangan Pahang Kampus Jengka, Bandar Tun Razak 26400, Pahang, Malaysia; 3 Institute of Tropical Forestry and Forest Products, University Putra Malaysia, Serdang 43400, Selangor, Malaysia; 4 Faculty of Forestry and Environment, University Putra Malaysia, Serdang 43400, Selangor, Malaysia; 5 Laboratory of Adhesive Sciences and Bio-Composites, Department of Biomaterial Sciences, Graduate School of Agricultural and Life Sciences, The University of Tokyo, 1-1-1 Yayoi, Bunkyo-Ku, Tokyo 113-8657, Japan; 6 Faculty of Forest Industry, University of Forestry, 1797 Sofia, Bulgaria

**Keywords:** polyol, NCO/OH ratio, FT-IR, 2D-COS analysis, bio-based PU

## Abstract

The compatibility between isocyanate and polyol plays an important role in determining a polyurethane product’s performance. This study aims to evaluate the effect of varying the ratios between polymeric methylene diphenyl diisocyanate (pMDI) and *Acacia mangium* liquefied wood polyol on the polyurethane film properties. *A. mangium* wood sawdust was liquefied in polyethylene glycol/glycerol co-solvent with H_2_SO_4_ as a catalyst at 150 °C for 150 min. The *A. mangium* liquefied wood was mixed with pMDI with difference NCO/OH ratios to produce film through the casting method. The effects of the NCO/OH ratios on the molecular structure of the PU film were examined. The formation of urethane, which was located at 1730 cm^−1^, was confirmed via FTIR spectroscopy. The TGA and DMA results indicated that high NCO/OH ratios increased the degradation temperature and glass transition from 275 °C to 286 °C and 50 °C to 84 °C, respectively. The prolonged heat appeared to boost the crosslinking density of the *A. mangium* polyurethane films, which finally resulted in a low sol fraction. From the 2D-COS analysis, the hydrogen-bonded carbonyl (1710 cm^−1^) had the most significant intensity changes with the increasing NCO/OH ratios. The occurrence of the peak after 1730 cm^−1^ revealed that there was substantial formation of urethane hydrogen bonding between the hard (PMDI) and soft (polyol) segments as the NCO/OH ratios increased, which gave higher rigidity to the film.

## 1. Introduction

Thermoplastic polyurethanes are one of the very flexible polymers, being employed in a wide range of industrial and engineering applications, such as automotive instrument panels, footwear, medical devices and highly demanding transparent film applications [1,2]. Their wide range of applications is related to the superior properties of the urethane linkages (NHCOO) in the molecular backbone, which are obtained via the reaction between isocyanate and polyol. Polyols are molecules consisting of multiple OH groups that correspond to the flexibility of the polymer, while isocyanate is characterized as the groups of NCO that contribute to the rigidity [2,3,4,5].

The use of liquefied wood in the production of polyurethane requires extensive investigations to determine the workable ratios between the liquefied wood and pMDI. These ratios are crucial in influencing the properties of the resulting films. The compatibility between polyol and isocyanate is typically measured based on the isocyanate index (NCO/OH). The isocyanate index refers to the amount of isocyanate used in relation to the theoretical equivalent amount and has a remarkable impact on the polyurethane structure and mechanical properties. High NCO/OH ratios usually enhance material rigidity and crosslinking density [1,3,6].

The preparation of PU from liquefied wood is not a new approach. Lee et al. [7] synthesized waterborne polyurethane film from liquefied lignin and found that the addition of liquefied lignin enhanced the rigidity of the films. Nevertheless, the complex structure of liquefied lignin means some components could not totally react with isocyanate and corresponds to the decrement of the PU film’s tensile properties [8]. Gosz et al. [9] blended pMDI with liquefied alder wood and liquefied oakwood at different solvent and different NCO/OH ratios. It was found that the formulation NCO:OH 1:1 containing liquefied wood with only PEG400 showed better thermal stability, which was due to the high content of high molecular weight PEG present in the liquefied wood. Lee and Lin [10] produced PU adhesive from Taiwan acacia and China fir liquefied wood and disclosed that the dry and wet bonding strengths of the wood specimens were enhanced concurrently with the increasing NCO/OH ratio. The wet bonding strength of the wood samples at 1.5 and 2.0 NCO/OH ratios were more than 5.9 MPa, even after immersion in water (60 °C), which is acceptable according to the CNS 113031 standard requirement. Jiang et al. [11] reported that the bonding strengths of wood bonded with crude liquefied wood polyurethane and pure liquefied wood polyurethane were enhanced by increasing the NCO/OH ratio from 0.5:1 to 1.5:1. However, the bonding strengths were slightly decreased after the ratio increased to 2:1, which is related to the wide distribution of the crosslinking density within the adhesive network due to the side reaction. PU film also has been prepared from sugi liquefied wood, where a high isocyanate index significantly enhanced the tensile strengths of the PU films [12]. The crosslink density and T_g_ of the films were enhanced due to the amount of dissolved woody components in the films, wherein the dissolved woody components in liquefied wood have many OH groups and act as crosslinking agents that integrate with isocyanate for PU network formation. Yao et al. [13] prepared rigid PU foam from PEG-liquefied wood and starch and found that the mechanical properties were almost similar to those of commercial products.

Lu and Larock [14] studied the effect of polyol functionality and hard segment content on soybean-oil-based waterborne polyurethane (SPU) dispersion and found the T_g_ value of the SPU films enhanced concurrently with the increasing polyol functionality and hard segments. Huang and Zhang [15] showed the glass transition temperature, the stiffness and tensile strength of nitrolignin polyurethane films increased with an increasing NCO/OH ratio. Patel and Mishra [16] observed that the thermal stability of di-phosphorous-based polyether ester urethanes was also improved for higher NCO/OH ratios. This behavior is related to the enhancement of the microphase separation. Higher NCO/OH ratios enhance the formation of urea, leading to a stronger intermolecular and intramolecular hydrogen bond, resulting in higher stiffness polyurethane [12,15,17,18,19].

In our previous study, *A. mangium* wood has been successfully liquefied in a PEG/glycerol solvents mixture with sulfuric acid as a catalyst at 150 °C in 150 min, which resulted in about 75% of *A. mangium* polyol [20]. The objectives of the study were to determine the feasibility of using *A. mangium* liquefied wood for the production of BIO-PU films and to elucidate its behavior as affected by the NCO/OH ratios, heating temperatures and durations. In this study, the *A. mangium* liquefied wood was polymerized with pMDI in the production of bio-based PU (BIO-PU) film, where the effects of varying ratios of NCO/OH on the BIO-PU film properties were investigated. The thermal, mechanical and solubility properties of the films were studied. The effect of the heating time was also evaluated since heat treatment was applied in the films’ formation. The effects of the inter- and intramolecular interactions on the BIO-PU films were investigated by means of FT-IR and further elucidated via a 2D-COS analysis.

## 2. Materials and Methods

### 2.1. Materials

The *Acacia mangium* wood chips were supplied by Sabah Softwood Berhad (SSB, Sabah, Malaysia). The wood chips were ground and sieved to obtain 20–100 mesh wood flour sizes. The polymeric methylene diphenyl diisocyanate (pMDI) (MR-200) was supplied by TOSOH Corp. The acetone was purified following the Almarego and Peerin [21] method. The methanol, toluene and *N*, *N*, dimethylformamide (DMF) were dried with a 4A molecular sieve before usage, and the other chemicals were reagent grade and used as received.

### 2.2. Liquefaction of Wood

About 10 g of *A. mangium* wood flour (20–100 mesh) was liquefied at 150 °C in 150 min using a mixture of polyethylene glycol (400) and glycerol (80:20 ratios) solvents in the presence of 3% *w/v* sulfuric acid. The liquefied wood was diluted with methanol and neutralized using 48% NaOH [12]. The solution was filtered using Kiriyama filter paper (5C) and the solid residue was removed, followed by an evaporation process at 45 °C, before being dried in a vacuum oven at 80 °C for 24 h. The water content of the liquefied wood was 2%, as measured using a moisture analyzer (MX-50, A&D Company, Limited, Tokyo, Japan).

### 2.3. Preparation of Polyurethane Film

The *A. mangium* polyurethane (BIO-PU) films were prepared by mixing the liquefied wood (hydroxyl number 458 mg KOH g^−1^) with pMDI (isocyanate content of 7.268 mmolg^−1^) at different NCO/OH ratios based on the formulation, as shown in Table 1. The hydroxyl number of the liquefied wood was calculated via esterification with phthalic anhydride and the isocyanate content was measured based on the back titration method (Machrom). The NCO/OH ratio was calculated by Equation (1) following [12]. In this study, four NCO/OH ratios of 0.5, 0.6, 0.7 and 0.8 were used to produce the BIO-PU films, which were designated as BIO-PU0.5, BIO-PU0.6, BIO-PU0.7 and BIO-PU0.8, respectively.

(1) $$\mathrm{NCO}/\mathrm{OH}\;\mathrm{ratio}=\frac{M_{\mathrm{pMDI}\;}\times W_{\mathrm{pMDI}}}{M_{\mathrm{polyol}\;}\times W_{\mathrm{polyol}}+W_{\mathrm{water}\;}\times 2/18\times 1000}$$

where *M*_pMDI_ is the isocyanate content (7.268 mmolg^−1^), *M*_polyol_ is the hydroxyl group of the liquefied wood (hydroxyl number/56.1, molg^−1^) and *W*_pMDI,_ *W*_polyol_*,* and *W*_water_ are the weights of pMDI, liquefied wood and water included in the liquefied wood. Moreover, 2/18 × 1000 represents (2 g water/18) × 1000 mL, while 18 is the molecular weight of H_2_O.

The liquefied wood was diluted with twice the amount of distilled acetone using ultrasonics for 60 s before vigorous mixing with the predetermined pMDI for another 20 s. The polymerized mixtures then were poured into a petri dish and left overnight in a dark place in order to control the moisture from the surroundings before being dried in a vacuum oven at 100 °C for 8 h. For the effect of time, the BIO-PU films with 0.6 NCO/OH (BIO-PU0.6) were prepared and dried in a vacuum oven (100 ± 5 °C) at different reaction times: 4 and 8 h.

### 2.4. Measurements

The FT-IR spectra were recorded on a Nicolet 6700 FT-IR Spectrometer (Thermo Fisher Scientific Corp, Yokohama, Japan). About 128 scans were accumulated at 4 cm^−1^ resolutions within the frequency range of 4000–1000 cm^−1^. A thin film of polyurethane was cast directly on a calcium fluoride (CaF_2_) disk and left overnight at room temperature. For the effect of NCO/OH, the cast film was dried in a vacuum oven at 100 ± 5 °C for 8 h and put in desiccator for 15 min before being analyzed. For the effect of time, the FT-IR measurement were taken every hour until 8 h. For the temperature-dependent study, the cast film was heated using an HT-32 Heated Demountable Cell as a temperature control during the FT-IR measurement. A second derivative technique and peak deconvolution were also applied using the OMNIC FTIR software for Nicolet FT-IR spectrometers. The best fit was obtained using the Gaussian–Lorentzian sum and the peak area was calculated after baseline correction. In this study, 2D-COS IR spectroscopy was used for the characterization of the structural changes in the PU films obtained as a function of different temperatures and NCO/OH ratios. The band of FTIR spectra at 2906 cm^−1^ served as the peak normalization and the resulting spectra were evaluated using 2DShige software (2D Shige (c) Shigeaki Morita, Kwansei Gakuin University, 2004–2005).

About 6–10 mg samples were analyzed on a PerkinElmer STA 6000 under an N_2_ atmosphere (20 mL min^−1^) with a heating rate of 10 °C min^−1^ in the range of 30 to 600 °C for the thermogravimetric analysis.

A dynamic mechanical analysis was carried out using a DVA-200s (ITK, Japan) with a heating rate of 10 °C min^−1^. The operating temperature ranged from −100 °C to 250 °C at a 10 Hz frequency in a dry nitrogen gas flow of 0.5 L min^−1^. Rectangular samples with a size of 10 mm in length and 4.5–5 mm in width were cut from the cast film. The glass transition temperature (T_g_) of the samples was obtained from the peaks of the tan δ curves. The crosslinking density was measured based on the DMA results following the kinetic theory of rubber elasticity (Equation (2)).

Storage modulus (Pa), E′ = 3 V_e_RT (2)

where V_e_ is the crosslinking density (mol cm^−3^), R is the gas constant (8.314 Jk^−1^mol^−1^) and T is the absolute temperature of rubbery plateau.

In the sol fraction test, about 0.1 g of BIO-PU films were placed in an oven at 105 ± 3 °C for 24 h before being immersed in *N, N,* dimethylformamide (DMF) for 3 days at 25 °C. About 4 replicates were prepared for each parameter. The swollen films were then removed from the solvent, blotted using tissue paper and dried at 105 ± 3 °C for another 24 h before being re-weighed. The percentage of soluble materials (sol fraction) was calculated using Equation (3).

Sol fraction (%) = (*W*_i_ − *W*_o_/*W*_o_) × 100 (3)

where *W*_i_ is the initial weight of the BIO-PU film after oven heating and *W*_o_ is the final weight of the BIO-PU film after the swelling test and oven drying.

## 3. Results and Discussion

### 3.1. Effect of Heating Time and Temperature

**Figure 1:**
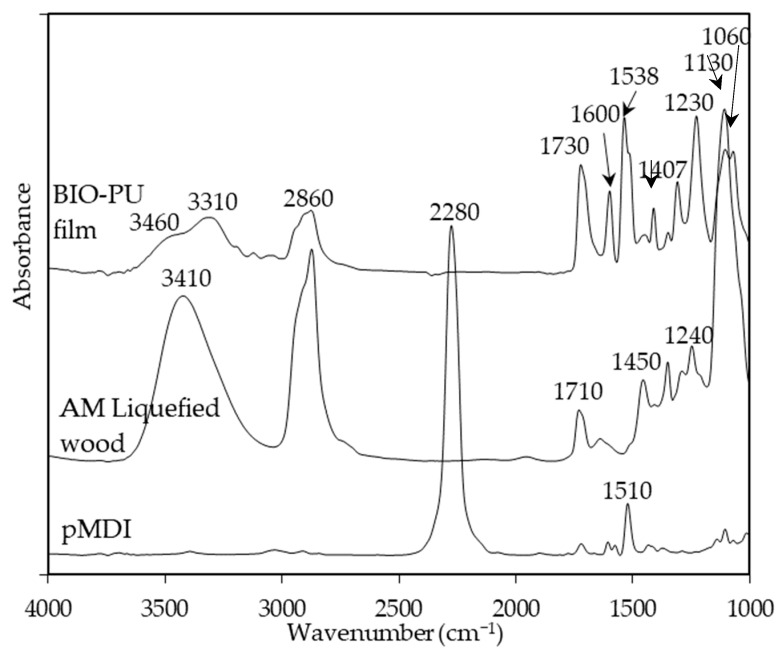
FT-IR spectra of BIO-PU film, AM liquefied wood and pMDI.

**Figure 2:**
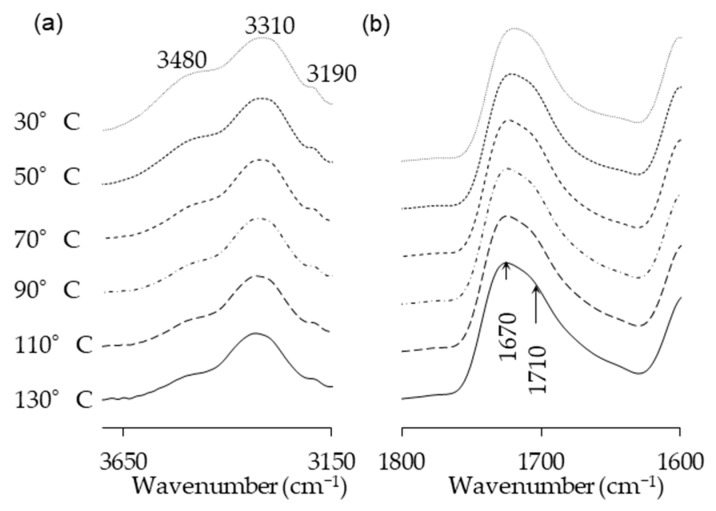
(**a**) N–H stretching and (**b**) amide regions of FT-IR spectra of BIO-PU0.6 at different heating temperatures.

**Figure 3:**
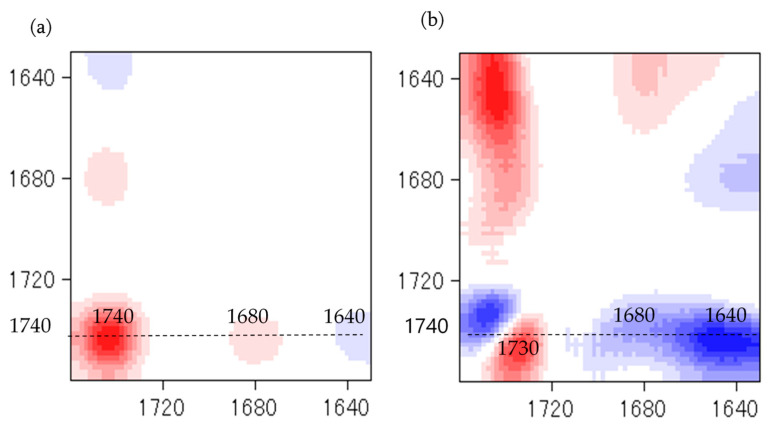
Synchronous (**a**) and asynchronous (**b**) spectra at different heating temperatures in carbonyl region.

Temperature is one of the main factors affecting the properties of PU film. In this study, the NCO/OH ratio 0.5 obviously shows a fairly short rubbery plateau, indicating a low distinct area of crosslinking density. In addition, the distinct and complete single tan δ peak (NCO:OH 0.6) indicates a homogeneous internal molecular structure in which stress concentration can be avoided and material can absorb more energy as a result of loading. Therefore, the BIO-PU 0.6 ratio was selected as a representative to determine the effect of heating on the BIO-PU film properties. The chemical structures of the BIO-PU film, AM liquefied wood and pMDI were analyzed via FT-IR spectroscopy (Figure 1). The existence of absorbance-related peaks with urethane linkages can be seen obviously in the absorbance bands at 3460, 3310 and 3290 cm^−1^, as attributed to the N–H stretching, 1730 cm^−1^ (C=O stretching), 1538 cm^−1^ (N–H bending) and 1230 cm^−1^ (C–N stretching) [12,22,23]. The arising peaks are attributed to the polyols located at 2840 and 2925 cm^−1^, which are associated with the symmetric and asymmetric C–H stretching vibrations [22], 1450 cm^−1^ (C–H bending), 1320 cm^−1^ (C–H rocking of the methyl group) and 1110 cm^−1^ (C–O–C stretching). The absorption bands at 1530 and 1410 cm^−1^ correspond to the C–C and C–H stretching aromatic ring of the pMDI, respectively. The temperature effects (30–130 °C) on the BIO-PU film formation were investigated (Figure 2). The spectra of the N–H stretching region (Figure 2a) show a strong absorption band centered on 3320 cm^−1^ that corresponds to the hydrogen bonding of NH to carbonyl groups [22,24]. As the temperature increases, the band at 3320 cm^−1^ shifts to a higher wavenumber and continuously decreases in the area. A similar spectral change was reported by Skrovanek et al. [25], Coleman et al. [22], Wang et al. [26], Teo et al. [24], Yilgor et al. [27], Xu et al. [28] and Jung et al. [29]. They found the reduction in the H-bonding strength to correspond to the transformation of the H-bonded N–H groups fraction to the non-H-bonded groups. According to Teo et al. [24], the strength of hydrogen bonding is deteriorated at temperatures of more than 50 °C, where the NH H-bonded to ether oxygens decreases more remarkably compared to the NH H-bonded to carbonyl groups. Meanwhile, the free NH stretching vibration appears as a weak shoulder located at 3460 cm^−1^. In addition, another obvious peak emerges at 3190 cm^−1^. Teo et al. [24] obtained the stretching vibration of the NH O–H-bonded at 3268 cm^−1^, which signifies there are a number of hard–soft segments H-bonded in their polymer’s samples. The heating temperature differences (30 to 130 °C) spectra in the carbonyl stretching region can clearly be seen in Figure 2b. As observed, the sample shows a very strong absorption peak at 1710 cm^−1^, attributed to the H-bonded urethane carbonyl groups [24,27,30]. As the temperature increased, the band at 1710 cm^−1^ altered to the higher frequency at 1730 cm^−1^ (free C=O) and decreased in the intensity of the band. This trend is like that of previous studies on polyurethanes [22,24,25,26,27,28]. Teo et al. [24] suggested a frequency shift is directly related to a decrease in the fraction of hydrogen-bonded urethane carbonyls with an increasing temperature. Interestingly, there is a distinct shoulder located between 1640 and 1670 cm^−1^ that increased as the temperature raised, indicating the formation of a strongly hydrogen-bonded urethane hard segment [19,22,24,27]. For a better understanding, the 2D-COS technique was used to comprehend the correlation among the functional groups in this region. It is very interesting to observe that only one strong autopeak can be seen in the synchronous spectrum (Figure 3a). According to Noda [31], a spectrum that changes its intensity greatly due to perturbation will show a strong autopeak, meaning that the band at 1740 cm^−1^ is very susceptible to the temperature in the carbonyl region. This is consistent with the trend of the IR peak, where the band at 1710 cm^−1^ (Figure 2b) shifted to a higher wavenumber as the temperature increased. The development of two cross peaks in the synchronous spectrum located at 1680 (H-bonded urethane and ordered C=O) and 1640 cm^−1^ (bonded C=O groups in the hard segment) demonstrated the formation of microphase separated and ordered urethane hard segments [27]. The concurrent increase in the intensities of 1680 cm^−1^ and 1740 cm^−1^ as the temperature increased is confirmed by the presence of a pair of positive cross peaks. The presence of negative cross peaks between 1640 cm^−1^ and 1740 cm^−1^ shows the intensity of 1640 cm^−1^ is reduced as the sample is heated. It can be concluded that the ordered urethane hard segment (1680 cm^−1^) was enhanced whilst the H-bonded urea (1640 cm^−1^) was reduced as the free C=O at 1740 cm^−1^ increased as a function of the temperature. In the corresponding asynchronous spectrum (Figure 3b), the presence of 1730 cm^−1^ reveals that 1740 cm^−1^ was split into 1730 cm^−1^. The similar sign (positive sign) of the 1730/1740 cm^−1^ for both the synchronous and asynchronous spectrums reveals that the 1730 band changed first followed by the 1740 cm^−1^. The negative sign between 1640/1740 (negative sign in the corresponding synchronous spectrum) shows that the 1640 occurred before the 1740 cm^−1^, whereas the negative cross peak at 1680/1740 (positive sign in the resultant synchronous spectrum) shows that the band at 1680 occurred after that at 1740 cm^−1^. Thus, the sequence motions for the cross-correlation in the carbonyl region can be drawn as follows: 1640→1740→1680 cm^−1^. The H-bonded urea (1640 cm^−1^) occurring first followed by the free urethane reveals that urea followed the first-order reaction [2]. The existence of urea linkages at 1640 cm^−1^ implies that a high temperature had spurred on the water and isocyanate interaction, leading to high urea linkage formation. The formation of the 1680 cm^−1^ peak, which occurred after the 1740 cm^−1^ peak, indicates the formation of a urethane hard segment [27].

**Figure 4:**
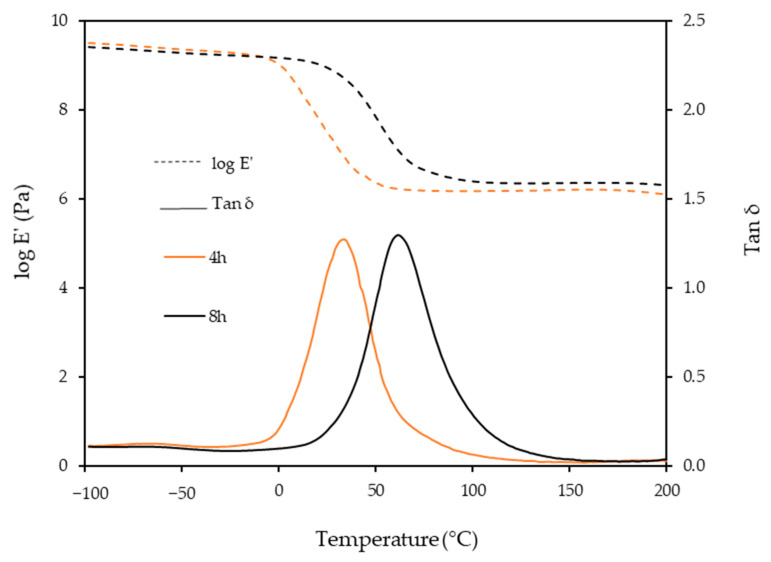
Dependence of storage modulus, E′ and tan δ of BIO-PU0.6 films on temperature with different heating times.

Figure 4 shows the storage modulus (E′) and tan δ of the BIO-PU films after heating at 4 and 8 h, respectively. The BIO-PU films are in a glassy state at temperatures below 0 °C, and it decreases slightly with the temperature. A rapid decrease in the E′ is observed in the temperature range between 0 and 100 °C, corresponding to the primary relaxation process of the resulting materials. The decrement of this modulus corresponds to an energy dissipation and the maximum is observed in the tan delta curve. A distinct shifting of glass transition (T_g_) from 35 to 62 °C occurs when the heating time is increased from 4 to 8 h, suggesting that the films become harder due to the restricted movement of the soft segment chains caused by the higher crosslinking [12]. In addition, the obviously greater enhancement in the rubbery plateau region for 8 h reveals that lengthening the heating time had induced net-points cross-linking, which leads a stronger intermolecular and intramolecular hydrogen bond.

### 3.2. Effect of NCO/OH Ratios on BIO-PU Film Properties

**Figure 5:**
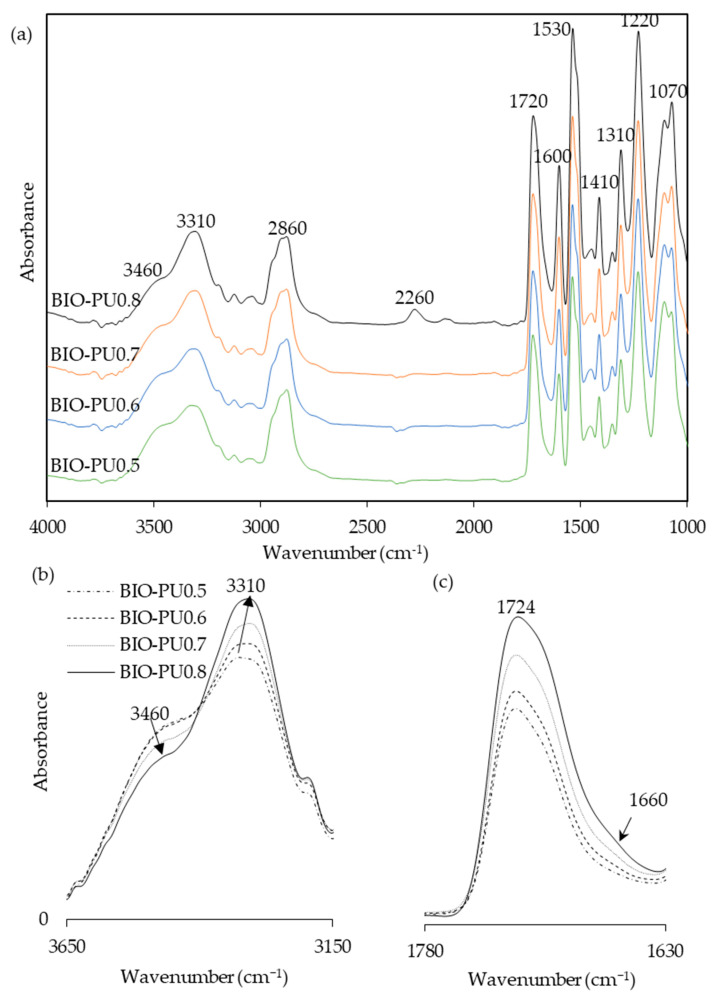
(**a**) FT-IR spectra, (**b**) NH-stretching region, and (**c**) amide region of BIO-PU films at different NCO/OH ratios.

**Figure 6:**
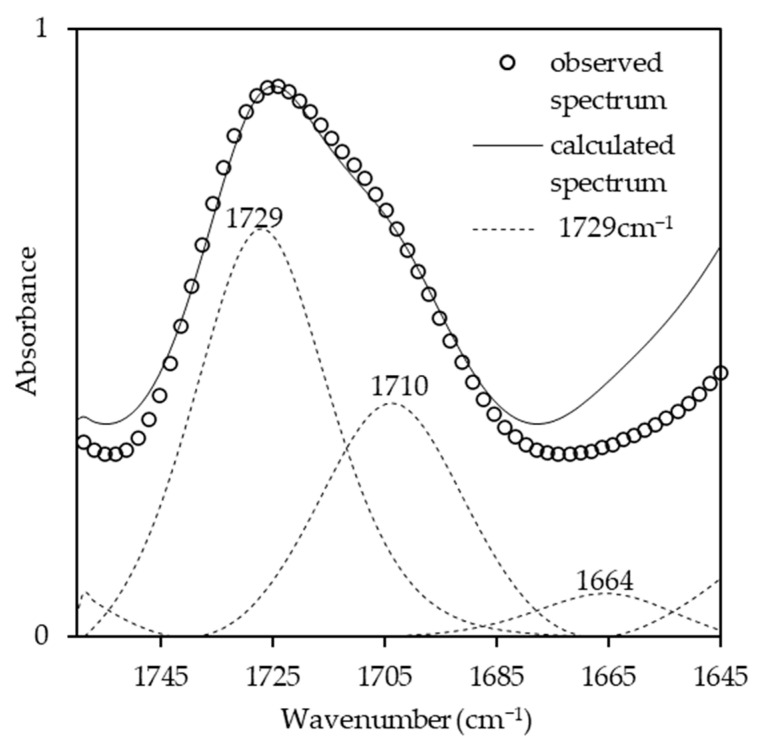
Curve-fitting results in carbonyl region of BIO-PU films.

**Figure 7:**
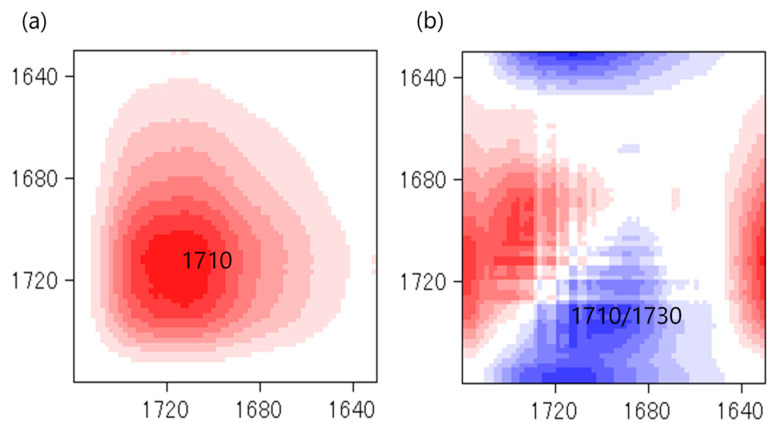
Synchronous (**a**) and asynchronous (**b**) spectra in carbonyl region at different NCO/OH ratios.

The effects of the NCO/OH ratios were studied by blending the AMpolyol with pMDI at different NCO/OH ratios (Table 2). The chemical structures of the BIO-PU films were analyzed using FT-IR spectroscopy. All the FT-IR absorbances were normalized at 2906 cm^−1^ (Figure 5a). Obviously, the broad (N–H) peak located at 3460 cm^−1^ reduced, became much sharper and shifted to 3310 cm^−1^ with an increasing NCO/OH ratio (Figure 5b), which is correlated with its tendency to form hydrogen bonds within hard segments [2]. The formation of amide groups was also enhanced, which is dependent on the NCO/OH ratio (Figure 5c). The non-hydrogen bonded C=O peak at 1730 cm^−1^ increased while the presence of an absorbance peak as a shoulder was enhanced in accordance with the NCO/OH ratio. The curve-fitting method was performed on the recorded spectra. Three Gaussian–Lorentzian sums were considered in the curve fitting of the carbonyl region (Figure 6) at 1729 cm^−1^, 1710 cm^−1^ and 1664 cm^−1^, where they corresponded to C=O free urethane, C=O H-bonded urethane and C=O urea [4], respectively. Distinctly, the formation of urea (1664 cm^−1^), which is not present initially (Table 2), becomes a stronger shoulder with an increasing NCO/OH ratio. The water molecules in the polyol reacted with the isocyanate and produced unstable carbamic acid before disintegrating into amine and carbon dioxide (CO_2_) [12]. Further reactions among the amine and another isocyanate led to urea formation. The formation of urea was also induced by the temperature (see Figure 3b). The integrated absorbance for all the bands increased with an increasing NCO/OH ratio. Accordingly, a 2D-COS analysis was used to investigate the formation of hydrogen bonds in the carbonyl region dependent on the NCO/OH ratio. The strong autopeak observed at 1710 cm^−1^ (Figure 7a) demonstrates that the C=O H-bonded urethane has the most significant intensity changes with an increasing NCO/OH ratio. In the corresponding asynchronous spectrum (Figure 7b), the band at 1710 cm^−1^ has been split into a 1730 cm^−1^ band. The negative sign of the cross peak at 1710/1730 shows a positive sign in the corresponding spectrum, reflecting the following sequence: 1730→1710 cm^−1^. It reveals that the absorbance band at 1710 cm^−1^ is enhanced depending on the NCO/OH ratio, and the changes of the 1710 cm^−1^, which occurred later, reveal that the C=O H-bonded urethane deviated in a slow process that was stimulated by the changes in the free urethane (1730 cm^−1^).

**Figure 8:**
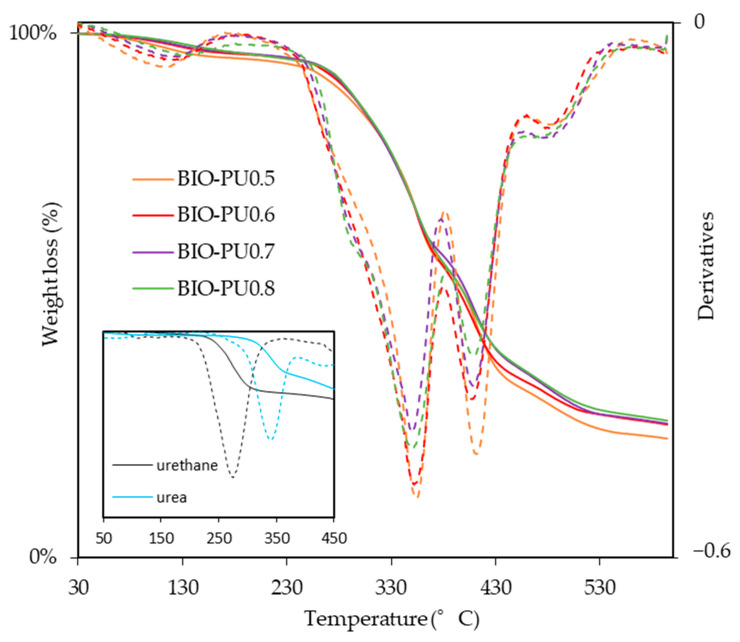
TG/DTG of BIO-PU films at different NCO/OH ratios.

The effects of the NCO/OH ratio on the TG curves of the BIO-PU films at the NCO/OH ratios of 0.5, 0.6, 0.7 and 0.8 are shown in Figure 8, and the corresponding data are summarized in Table 3. The derivative of the weight-loss curves shows the existence of five thermal degradation curves pinpointed at ca. 110 °C, 280 °C, 350 °C, 410 °C and 480 °C. The initial mass loss around 70–160 °C is related to the dehydration of the solvent and entrapped moisture in the film. The temperature of 10% weight loss showed the thermal degradation depending on the NCO/OH ratio, and a higher temperature is required to obtain the 10% weight loss of BIO-PU0.8 compared to the other ratios. The degradation processes in this temperature range of 270 to 300 °C correspond to the urethane linkages, which is also illustrated in the inset. The decomposition step, which is derived from urea, happened in the third stage ca. 350 °C. Urea has a higher degradation temperature (340 °C) than urethane (270 °C) (inset in Figure 8). This is also supported by Long and Pisney [32], who state that urethane decomposition occurs between 290 and 370 °C, as observed via DSC. The fourth degradation stage occurs between 380 and 460 °C, corresponding to polyol. Petrovic et al. [33] reiterate that the dissociation of polyol is higher compared to the urethane bond. Markedly, BIO-PU0.5 which high polyol content shows a broad curve in this decomposition stage. Eventually, the high degradation ca. > 450 °C might be related to the isocyanurate linkages. The presence of excess isocyanate in the BIO-PU films might lead to isocyanurate linkage formation. The isocyanurate group is the most thermal stable structure in the polyurethane system, which is typically induced by trimerization among isocyanate at high temperatures [5,12,34].

**Figure 9:**
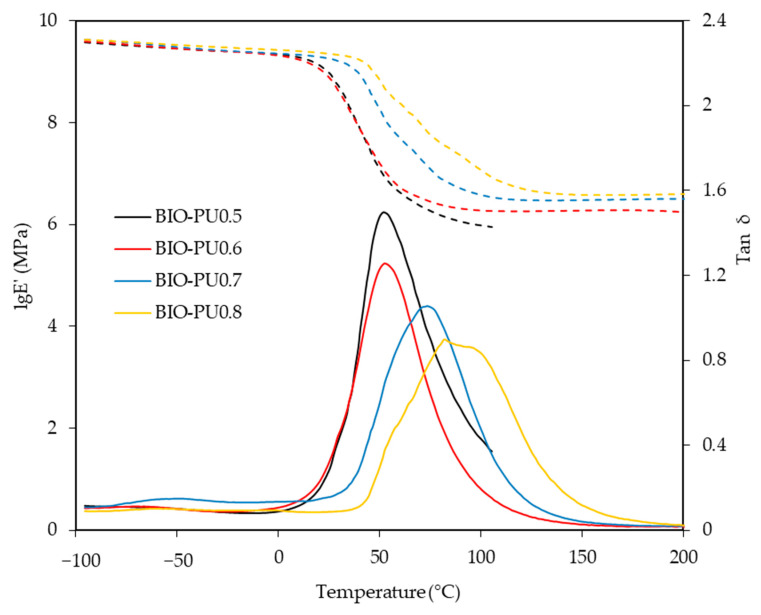
Storage modulus (dotted line) and tan δ (solid line) of BIO-PU films at different NCO/OH ratios.

**Figure 10:**
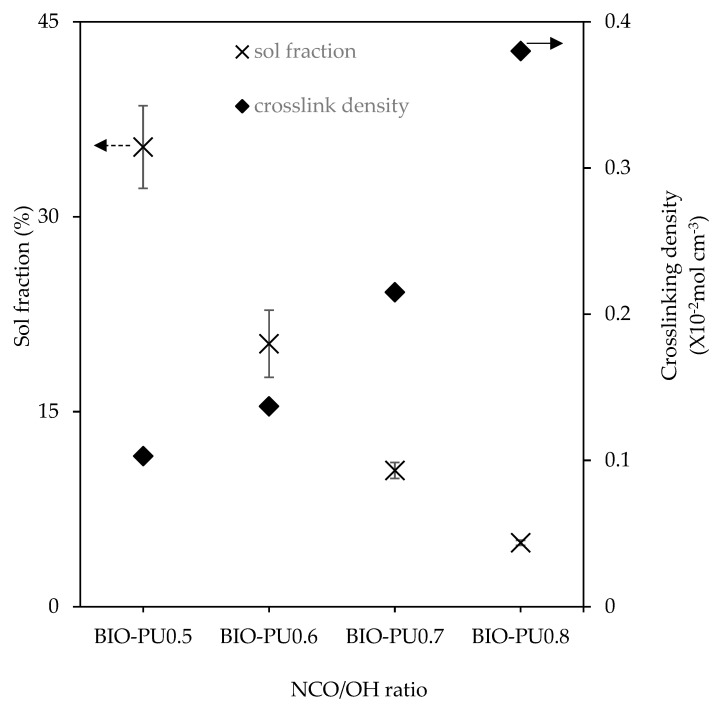
Crosslinking density and sol fraction of BIO-PU films at different NCO/OH ratios (n = 3).

The storage modulus (E′) and loss tangent (tan δ) of the BIO-PU films at different NCO/OH ratios as a function of the temperature are presented in Figure 9. Noticeably, the glass transition (T_g_) of the BIO-PU films became broadened and shifted to a higher temperature as the NCO/OH ratio increased, indicating the decrement of the polymer’s chain flexibility. This might be due to the improvement in the microphase separation in the copolymers with the increasing NCO/OH ratio leading to the formation of stronger hydrogen-bonded urea and urethane hard segments in the network. In addition, the temperature-insensitive rubbery plateau extending from 5 °C to around 200 °C indicates the presence of very strong hydrogen bonding and the well-separated microphase of the hard segment [27]. This is confirmed through the crosslinking density measurement, where the crosslinking density of the BIO-PU films is enhanced with an increasing ratio from 0.13 × 10^−2^ mol/cm^3^ to 0.38 × 10^−2^ mol/cm^3^ whilst the sol fraction is reduced significantly from 35.4% to 4.9% (Figure 10). The sol fraction is excluded from incorporation into the network formations of the films [12]. Thus, the reduction of the sol fraction concurrently with the NCO/OH ratio implies that a high NCO/OH ratio significantly enhances the interconnectivity among the hard segment domains where the incorporation of a urea hard segment can form extremely strong bidentated hydrogen bonding [27] and, consequently, reduce the solubility of the film. BIO-PU0.5 displayed only a fairly short rubbery plateau. The moduli of the rubbery plateau for BIO-PU 0.6–0.8 were around 10′ MPa. The data are presented in Table 4.

## 4. Conclusions

Prolonging the heating time considerably boosted the formation of urethane linkages. The T_g_ of the BIO-PU film shifted to a higher temperature after 8 h heating time, reflecting the improvement in hardness. The formation of urea linkages at 1640 cm^−1^, which occur earlier as revealed by the 2D-COS study, suggests the significant side reactions between isocyanate and water at a high temperature. For the effect of the NCO/OH ratio, the increment of the absorbance band at the N–H hydrogen bonded (3310 cm^−1^) depending on the NCO/OH ratio indicated a higher content of hydrogen-bonding interaction within the hard segment in the BIO-PU films. From the 2D-COS observation, the hydrogen-bonded carbonyl (1710 cm^−1^) had the most significant intensity changes with an increasing NCO/OH ratio, and its occurrences after 1730 cm^−1^ revealed that a high NCO/OH ratio induced the increment of urethane hydrogen bonding between the hard and soft segments. In addition, a high NCO/OH ratio significantly enhanced the hard segment formation, consequently boosting the crosslinking density of the BIO-PU films, which finally resulted in a low sol fraction.

## Figures and Tables

**Table 1:** Ingredients for BIO-PU film formation.

BIO-PU Film	Formulation (wt%)		NCO/OH Ratio
	*A. mangium* Liquefied Wood	pMDI	
BIO-PU 0.5	59	41	0.5
BIO-PU 0.6	54	46	0.6
BIO-PU 0.7	50	50	0.7
BIO-PU 0.8	47	53	0.8

**Table 2:** Characteristics of the urethane (free and H-bonded) of BIO-PU films in carbonyl region.

NCO/OH	Urethane (Free)		Urethane (H-Bonded)		Urea	
	Freq (cm^−1^)	Width ^a^ (cm^−1^)	Freq (cm^−1^)	Width (cm^−1^)	Freq (cm^−1^)	Width (cm^−1^)
BIO-PU0.5	1729	6.476	1710	7.176	-	-
BIO-PU0.6	1727	11.261	1703	7.940	1664	1.500
BIO-PU0.7	1729	8.835	1709	10.138	1666	1.677
BIO-PU0.8	1729	10.729	1707	11.956	1667	2.466

^a^ Width at half height.

**Table 3:** Thermal degradation stability of BIO-PU films.

BIO-PU Film	T_10wt.%_ (°C)	T_50wt.%_ (°C)	Residue at 600 °C (wt%)
BIO-PU 0.5	275	399	2.43
BIO-PU 0.6	283	396	2.57
BIO-PU 0.7	284	402	2.74
BIO-PU 0.8	286	400	2.71

**Table 4:** Glass transition (T_g_) temperature and storage modulus of BIO-PU films.

BIO-PU Film	T_g_ (°C)	E′ (MPa)
BIO-PU 0.5	50.15	8.32
BIO-PU 0.6	51.45	11.1
BIO-PU 0.7	72.25	18.2
BIO-PU 0.8	84.85	33.4

## Data Availability

The data presented in this study are available upon request from the corresponding author.
